# Factors that influence transition to advanced roles by RN to BSN nurses, in three selected hospitals of Central-Uganda

**DOI:** 10.1186/s12912-019-0345-z

**Published:** 2019-05-06

**Authors:** John Baptist Asiimwe, Mercy Muwema, Karen Drake

**Affiliations:** 1 Uganda Christian University, P.O. BOX 04, Mukono, Uganda; 2 Bishop Stuart University, P.O.BOX 09, Mbarara, Uganda; 3 Bethel University, 3900 Bethel Drive, St Paul, Minnesota 55112 USA; 4 Uganda Nurses, and Midwives Council, Kampala, Uganda

**Keywords:** Factors, Successful role transition, Advanced roles, RN to BSN nurses

## Abstract

**Background:**

Despite the global rise in the number of nurses upgrading from Registered Nursing (RN) to a Bachelor of Science in Nursing (BSN), studies have indicated that successful role transition is difficult once the nurses return to their previous workplaces. Guided by the Transitional Theory, this study investigates the factors that influence the transition from basic to advanced roles among RN to BSN nurses in Uganda, Africa.

**Methods:**

This study employed a descriptive correlational design. Using convenience sampling, fifty-one (51) RN to BSN nurses completed the semi-structured questionnaires.

**Results:**

All the study participants (100%) described themselves as having transitioned from RN to BSN role. In bivariate linear regression, personal factors that were found to predict successful role transition included holding a managerial role, being aware and prepared for the role transition, and positive role transition experiences. Role transition motivators that predicted successful role transition included: job promotion, internal desire for self-development, and career development. One community factor – that is the support of doctors/physicians during the RN to BSN transition – predicted unsuccessful role transition. Societal factors deterring successful role transition included lack of support from other colleagues and the perception that BSN learning was not applicable to the RN clinical setting. In multivariate linear regression, only sub-scales of personal factors such as advanced skills mastery and positive personal experiences predicted successful role transition.

**Conclusion:**

The study suggests that personal factors influence successful role transition more than external factors.

**Electronic supplementary material:**

The online version of this article (10.1186/s12912-019-0345-z) contains supplementary material, which is available to authorized users.

The focus of the study is on factors that influence successful transition from basic roles (diploma-RN roles) to advanced roles (BSN-RN roles) among registered nurses (RNs) who previously held a diploma but upgraded and acquired a degree in nursing (referred to as Bachelor of Nursing Science in Uganda and Bachelor of Science in Nursing elsewhere). The term RN to BSN in this article means registered nurses and midwives who previously held a diploma but upgraded and acquired a BSN. This study group was registered with the Uganda Nurses and Midwives Council (as RNs with BNS) and had returned to their previous workplaces.

## Background

Studies in North America indicate that BSNs are contributing to improved access to health services and better health outcomes. Notably, acute care hospitals with higher proportions of nurses educated at the baccalaureate level or higher, have been found to experience lower overall mortality rates by 5% in surgical patients who experience serious complications [1]_._ Recent studies have also reported a positive association between the BSN prepared nurses and professional behaviors vital to patient care (such as critical thinking, professionalism, and creativity) and patient safety [2, 3].

The increase in nurses transitioning from RN to BSN has been documented more in developed countries such as those in North America than in developing countries like Uganda. For instance, there was a 6% (32 to 38%) rise in the number of RN’s who obtained a BSN in the United States of America between 2005 and 2014 [2, 3]. In Africa, one study indicated that due to increased private sector interest and investment in nursing education, 32% of the sub-Saharan countries had established the bachelor of nursing program by 2005 [4, 5]. However, comprehensive data on the output of RN to BSN from the training institutions, in sub-Saharan Africa (including Uganda), is difficult to obtain [5].

Although little research was accessed on why RNs upgrade to BSN in sub-Saharan Africa, several studies, in developed countries point to more push factors than pull factors as motivators to RN to BSN transition [6, 7]. The push factors relate to the negative perceptions towards the RN roles. Such factors include limited opportunities for career advancement, lack of professional development, and a decline in employment opportunities for RNs among others. Other push factors were due to the global and local governmental and institutional shift in policies. Such policies include phasing out of the RN nurse roles, recognition of the RNs previous training by universities, and recognition by some countries/employers of the importance of having RNs with a BSN in the clinical setting [6–10]. In the United States, for example, the Institute of Medicine recommended that hospitals should develop plans to have 80% its RN staff possesses a BSN by 2020 [11, 12]. Meanwhile, pull factors are the positive inducements related to obtaining a BSN, such as, an expectation of an improved pay, personal confidence building, intrinsic drive for self-development, achieving goals of working with higher and increased responsibility as well as advancing careers [6]. Clearly demarcated roles of a BSN as perceived by RNs were also found to be a driving factor in the RN to BSN transition [6, 13].

There is a significant transition for the upgraded BSN nurses on return to practice as an RN in Uganda. During this transition, like in the developed countries, the RN to BSN nurses must translate their new learning into diverse practice settings and assume new roles. Unfortunately, upon returning to their former clinical workplaces, they found the roles of an RN with a BSN difficult, broad, complex, and more mentally, and physically challenging than they would have expected [7–9]. Partly, the broadened scope of practice of the BSNs and the feeling of pressure from assuming more authority, increased accountability, and responsibility accounted for difficulties in adjusting to their new roles [6, 7, 9]. In addition, the RN to BSN nurses were sometimes not allowed to practice their new roles. Other times, they practiced both RNs and BSNs roles (dual identity), and due to this dual identity, they did not delegate duties that they previously performed as RNs [8, 9]. Above all, the RN to BSN nurses were found not prepared for those roles or the role transition process and their previous knowledge of the complexity of the clinical setting did not make their transition easier [6, 8, 10, 14].

Nayda & Cheri [10], mention four factors that may influence a successful transition after the BSN for a nurse with an RN background. These include: 1) the impact of workforce/place reality; 2) the available support systems; 3) knowledge, and skills expected of an RN; and 4) adjusting to the levels of accountability and responsibility. Indeed, factors within RN to BSN role transition experiences (such as barriers and support mechanisms) often had a significant effect on the successful role transition [7, 8, 10, 15]. In fact, those who obtained support during RN to BSN role transition felt it was very vital, such as when some physicians (clinical doctors) taught the new BSN skills when they observed that they were not confident [6]. However, the majority of the studies retrieved reported lack of support/mentoring given to the new BSNs, by either the employing organizations, management, peers or other health professionals, which led to a reduction in confidence while settling into the BSN roles, and consequently struggling to master those advanced roles [8, 10, 15].

Unfortunately, among the studies retrieved, there was minimal quantitative information on what factors influence a successful role transition from RN to BSN [7–9]. In addition, there has been a call for research on why some people (including nurses) transition well into the new role and others don’t; the influence of personal characteristics (such as age) on transitional experiences; how intrapersonal factors affect transition experiences; and the cultural experiences of transition [16].

For a long time in Uganda, like other parts of Africa, RNs were trained in institutions attached to hospitals and they acquired a diploma in either Nursing, or Midwifery, or both [17]. After Uganda’s independence in 1962, being an RN was the highest level of education that a nurse/midwife could attain. As an RN then, the nurses were expected to perform their roles under the physicians’ (doctors’) order with little independence [17]. In 1993, the first BSN course was introduced in Makerere University (Kampala, Uganda), with an expectation that the nurse leaders who were BSN graduates would increase public respect for the nurses’ work and provide a leverage to increase the independence of the nurses from the dominance of doctors in hospitals and health centers [17]. Whether this broad goal has been achieved remains a debate, but what is known is that since then, there has been an increasing shift from the hospital-based training of nurses to universities and thus many RNs have been able to acquire the BSN degree.

In Uganda, like in Europe, Oceania, and America, BSNs upon graduation should have mastered certain advanced skills with the expectation to use those skills. Among them, BSNs are expected to be rational with a higher degree of independence, and should be able to use decision-making skills, critical thinking skills, research evidence skills to better patient outcomes, and managerial skills [18]. BSNs should also be able to assume more responsibility and authority over patient care. Furthermore, the main difference in roles performed by BSN workers from those of an RN is that, apart from the basic roles that are performed by all RNs, the depth of performance of those roles among BSNs varies dramatically. For instance, among other roles, BSNs are supposed to perform a comprehensive health assessment as compared to a basic health assessment, use nursing theories, and utilize a broad knowledge base to inform care plans, conduct research to provide evidence to health problems, and are to report and make suggestions on patients’ conditions to doctors/physicians. The comprehensive performance of those roles is rare among RNs only because of the depth of the training curriculum, which places less emphasis on knowledge and attitude components, but give priority to the skills’ aspect [17, 19, 20].

This study expected that in the performance of the advanced roles, the BSN nurse develops a holistic, individualized patient care plan, based on a comprehensive assessment and implements this plan in consultation with other health care professionals and available research evidence (with a greater degree of independence). The BSN nurse also should be able to perform the advanced roles confidently, comfortably, and competently; receive the support from colleagues that enabled him/her (BSN) to execute those roles; and have their roles recognized by the other staff and their clients/patients [21]. Meanwhile, basic roles as performed by RNs relate to the performance of all nursing activities that are geared towards providing general nursing care to all patients as a whole and usually under orders of a senior nurse or doctor (minimal independence).

Similarly, evidence also indicates that RN to BSN graduates view a familiar situation in different ways and demonstrate those perspectives in new and different approaches to patient care [3]. However, experiences in Uganda were that upon return to their previous workplaces, some RN to BSN nurses were finding it difficult to change the way they practice their roles even after acquiring a BSN. Even those who initially adopted these new ways of practicing their roles later regressed back to practice those roles just as they did as RNs (personal communication with health managers at Regional Referral Hospitals, January 2016). It was noted earlier that many health workers in Uganda (RN to BSN inclusive) were not up to expected standards after returning from their training [22]. Besides that, nurses in Uganda, like those in other Sub Saharan Africa have an added burden of taking up extra tasks/roles, due to the shortage of other professional staff, and thus making their roles ambiguous and comparable to a nurse practitioner’s role in Europe and North America [23]. Patients, politicians, and nurses themselves were also dissatisfied with the quality of care services provided [17]. Additionally, those observations by health managers had further implications on the nursing image and investment in nursing education, which are still poor in Uganda [17].

From a theoretical perspective, the Transitional Theory postulates that all transitions involve change and that towards the end of transitions there is mastery of certain skills meant to help one to manage the new state(s) [16]. Thus, mastery of those skills is an indicator of a successful transition-outcome. However, the process and outcome of the transitions are facilitated or inhibited by the personal, community, and societal factors. Further, the key to a successful transition lays in prior preparation and having knowledge on what to expect during role transition, receiving community support during the difficult part of the transition, and being free from societal conditions (barriers) that may impede transitions.

Unfortunately, in Uganda (a Sub-Saharan country), there was little accessible data on the proportion of RN to BSN nurses who had mastered the advanced skills of a BSN and the proportion of RN to BSN nurses who had transitioned to advanced roles of the BSN, and the factors that influenced that successful role transition. Therefore, this study sought to answer the following research question: “What factors influence transition from the basic RN nursing roles to the advanced roles of the BSN nurse?”

Knowing the factors that influence the transition to advanced roles would eventually help the health leadership (nursing inclusive) understand the strategies that could be employed to assist RN to BSN nurses’ role transition successfully at their respective workplaces. This study envisaged that laying strategies that address the factors that influence the RN to BSN role transition in Uganda would in the short-term improve the quality of health care provided to the patients and the independence, and professionalism of BSN nurses. In the medium term, this would improve patient safety, other patient health care outcomes, and access to health care services. Subsequently, this would have a positive impact on the nursing image in Uganda and improve investment in nursing education by government and other stakeholders, reformation of the health care system, and human resource retention in public hospitals (thus reducing investment in recruitment and capacity building).

## Methods

### Study setting

The study was carried out in three large public and national referral hospitals, located in Central Uganda. These Hospitals were selected as study sites because they employ a large number of BSN nurses in Uganda (MOH, 2016 records).

### Research design, sampling, and sample size

This study used a descriptive correlational design. Approximately 60 nurses and midwives in total, who had transitioned from RN to BSN from the three hospitals, formed the sampling frame. Using convenience sampling, fifty-one (51) RN to BSN nurses (85% response rate) agreed to participate in this study. The 15 % (15%) non-response rate was due to failure to return the questionnaires’, refusal to consent, and some study participants had left the study settings.

### Selection criteria

The study included RN to BSN nurses who were registered with the Uganda Nurses and Midwives Council as RN (BSN) nurses. The RN to BSN nurses had to have completed their nursing education in Uganda (diploma and degree) and practiced in the Ugandan healthcare system (as RNs and as BSNs). In addition, those who possessed at least six (6) months of clinical experience after BSN were selected to participate in this study.

### Data collection procedure

Data was collected for over a six (6)-week period between January 15 to March 1, 2017. After the university and hospital approvals, RN to BSN nurses were invited to participate in the study, which took about 15 to 30 min.

Measurement of Variables (Additional file 1).

### Dependent variables

#### Mastery of advanced skills

Six items were constructed to measure the various areas of advanced skills or knowledge that are known to be key outcomes of the BSN program and are key for one to successfully execute his/her advanced roles (communication skills, leadership skills, critical thinking skills, research skills, pro-activeness, and knowledgeable). On a point, Likert scale (*1 = Strongly Disagree to 4 = Strongly Agree*) respondents were asked to rank their level of agreement with mastery of those skills. Higher scores were interpreted as a higher likelihood of advanced skills mastery and vice versa.

#### Role transition

Drawing from the general roles of RN to BSN as indicated in the literature, the newly developed, Ugandan nurses’ scope of practice [20], and the themes from the final nurse practitioner role transition scale [21], an RN to BSN role transition scale was created. The role transition scale (13 items) was computed from three subscales that measure support (1 item); BSN advanced roles and confidence, comfort, and competence of performing those roles today (10 items); and recognition of their roles by their clients and staff (2 items). A higher transition score was interpreted as a higher likelihood of a successful/ healthy transition and vice versa.

### Independent variables

#### Personal factors

##### Demographic characteristics

Eight (8) items measuring demographic characteristics were constructed. This encompassed gender, age (in years), experience (in years), and unit of work and registration status.

##### Motivators to pursue BSN

On a 4-point Likert scale (1 *= Not Motivating, 2 = Somewhat Motivating, 3 = Highly Motivating*), eighteen (18) items were constructed from the various reasons why nurses choose to pursue a BSN as reported in the literature. Such as job promotion, internal desire for self-development, career development, improved pay, job security, the presence of scholarships, competition from newer younger graduates, a decline in employment opportunities among diploma RNs’, and frustration with diploma RN roles among others.

##### Preparation and knowledge

On a 4-point Likert scale (*1 = Strongly Disagree, 4 = Strongly Agree*), two (2) items were constructed to establish if the respondent were knowledgeable and prepared for the RN to BSN transition just before resuming work as BSNs.

##### Cultural beliefs and attitudes

On a rating scale of 3 (*1 = Little/No Influence, 2 = Moderate Degree of Influence, and 3 = High Degree of Influence,),* two (2) items were constructed to establish if the culturally ingrained perception of the role of the nurse did actually have influence on the RN to BSN transition (Such as a perception that the nurses were doing doctor’s work and failure to obtain respect from other nurses).

##### Experiences

To establish if the respondent had an easy or difficult transitional experience, seventeen (17) items on a 4-point Likert scale (*1 = Strongly Disagree, 4 = Strongly Agree*) were constructed as reported in the literature. The experiences included an experience of job pressure, dual identity, guilt, emotional and or psychological dissatisfaction, and distress, role transition easiness related to previous experience, role ambiguity/confusion, support, and achievements of transition goals among others.

#### Community conditions (factors): support

On the rating of 3 (*1 = Little/No Assistance, 2 = Moderate Amount of Assistance 3 = Much Assistance*), six (6) items were constructed around the forms and the sources of support that RN to BSN received during their role transition.

#### Societal conditions (factors): barriers

Twenty-three (23) items on a rating scale of 3 (*3 = High Degree of Influence, 2 = Moderate Degree of Influence, and 1 = Little/No Influence*) were constructed around barriers that influenced RN to BSN transition upon resuming work. The barriers included lack of scope of practice, lack of promotion, the class teachings not applicable to the clinical setting, being few BSNs, high patient to nurse ratio, lack of support, and lack of equipment and logistics to use during care among others.

### Data analysis

Data were analyzed using SPSS (version 20, Additional file 2). Preliminary analyses were undertaken which included checking for outliers and missing cases, the normality of continuous data, reversal of negative items, and reliability analyses. Shapiro-Wilks test for normality was found to be 0.132, an indication that the total role transition scores (outcome) were normally distributed (*p* > 0.05). In addition, visual inspection of the histogram, normal q-q and box plots showed that the total role transition scores were approximately normally distributed, with a Skewness of − 0.32 (SE = 0.33), *z* = −.96 and kurtosis of − 0.786 (SE = 0.65), *z* = − 1.21. Under univariate analysis, frequencies and percentages were calculated for each of the categorical variables and means, standard deviations, or median, and ranges were also calculated for each of the continuous variables.

In bivariate analyses, Pearson Product-Moment Correlation Coefficient (*r*) was used to establish the relationship between the personal, community, and societal factors and successful role transition. Correlations of < 0.3, 0.3–0.4, and > 0.4 were considered weak, moderate, and strong respectively [24]. Second, bivariate or simple linear regression analyses were conducted to establish the explanatory and predictive power of each variable on successful role transition scores. In multivariate analyses, standard multiple linear regression was conducted on total scores of the independent variables that correlated with successful role transition. The level of statistical significance was established at an alpha level of 0.05 and 95% confidence intervals.

### Rigor

Prior to data collection, semi-structured questionnaires were pretested with seven (7) RN to BSN Nurses, in a Regional Referral Hospital located in Southwestern Uganda. Internal consistency was evaluated using Cronbach alpha (α) for each of the seven subscales (Table 1). Item analyses were also conducted testing each item’s relationship with other items (inter-item correlations-IIC) and with the total item scores (corrected-item total correlations-CITC). In summary, findings of this study were based on 88 items that were internally consistent with each other, with an overall Cronbach alpha of 0.762, the mean inter-item correlations of 0.044, and 22 items had corrected-item total correlations that were above 0.3 (Table 1).

**Table 1:** Internal consistency and measurement properties of items

Subscales	Items (N)	MIIC	Cronbach’s α	NCITC > 0.3
Overall	88	.044	.762	22
Motivators	19	.094	.677	9
Knowledge and Preparation	2	.690	.815	2
Experiences	20	.173	.807	15
Barriers	23	.200	.851	18
Support	6	.421	.816	6
Mastery	6	.421	.811	6
Role Transition Scale	13	.201	.718	9

*Note*. Mean inter-item correlation (MIIC), number of items with corrected-item total correlation (NCITC) > 0.3

## Results

### Research participants’ profile

The entire sample (*N* = 51) comprised of mostly female participants (74.5%). The majority of the participants were aged between 28 and 47 years (95.8%) with the mean age being 35.27 (±5.388 SD; Median = 35, Range = 27–57) years. The single trained nurses (74.5%) constituted the largest portion of the sample. Eighty percent (80.4%) of the participants had a previous RN experience ranging from 0 to 10 years (sample Mean of 7.63 ± 3.85SD, Median = 7, Range = 1–18). Similarly, the majority of the participants (86.3%) had an experience of 6 months to 10 years after completing the BSN (sample Mean of 2.89 ± 1.98SD, Median = 2, Range = 0.8–10). Interestingly, 66.7% of the participants were deployed on the same units that they were on prior to enrolling for the BSN. Nearly half of the participants (42.6%) were executing managerial roles on top of their patient care roles (Table 2).

**Table 2:** Participants’ profile

Variables	*n* (%)
Gender	
Male	13 (25.5)
Female	38 (74.5)
Age	35.27 ± 5.388^a^
18 – 27 Yrs.	1 (2.1)
28 –37 Yrs.	30 (62.5)
38 – 47 Yrs.	16 (33.3)
≥ 48 Years	1 (2.1)
Previous Training before BSN	
Nurse (Single Trained)	38 (74.5)
Midwife (Single Trained)	5 (9.8)
Nurse-Midwife (Double Trained)	8 (15.7)
Previous RN Experience	7.63 ± 3.85^a^
0 – 5 Yrs	20 (39.2)
6 – 10 Yrs	21 (41.2)
> 10 Yrs	10 (19.6)
Working Experience after BSN	2.89 ± 1.98^a^
6 months-5 Yrs.	28 (54.9)
6 – 10 Yrs.	16 (31.4)
> 10 Yrs.	7 (13.7)
Current Unit of Work	
Pediatrics	9 (17.6)
Obstetrics	4 (7.8)
Medical	10 (19.6)
Surgery	15 (29.4)
Psychiatry	1 (2)
Special Clinics (Heart, ICU, Cancer)	10 (19.6)
Any Other	2 (3.9)
Same Unit of Work before and after BSN	
Yes	34 (66.7)
No	17 (33.3)
Managerial Role (after BSN)	
Yes	20 (42.6)
No	27 (57.4)

*Note.* ^a^Mean ± standard deviation

### Advanced skills mastery

The proportion of participants who perceived to have mastered the advanced skills of a BSN was 98% (see Table 3). On univariate analysis, the largest proportion of participants (98%) reported having acquired more communication skills, research skills, and knowledge in patient illnesses/care issues than before the BSN. Meanwhile, the lowest proportions of participants recorded having acquired/possessed leadership (84.3%) and critical thinking skills (94.1%).

**Table 3:** Proportion of participants who perceive to have mastered the advanced skills

Variables	Yes *n* (%)	No *n* (%)
Overall	50 (98.0)	1 (2)
Leadership Skills	43 (84.3)	8 (15.7)
Pro-activeness	49 (96.1)	2 (3.9)
Communication Skills	50 (98.0)	1 (2)
Research Skills	50 (98.0)	1 (2)
Critical Thinking	48 (94.1)	3 (5.9)
Knowledgeable	50 (98.0)	1 (2)

### Role transition

All the study participants (100%) described themselves as having transitioned successfully from RN (basic) to BSN (advanced) roles (Table 4). Further univariate analysis indicated that, the largest proportion of participants reported working interdependently with other staff (100%), were performing their skills at a higher level (100%), and were more confident (98%), comfortable (96.1%), and competent (90%) than before when they were RNs only. The participants also reported that they were making independent rational decisions within their perceived scope of practice (78.4%) and 82.4% of the participants made patient care decisions independent of the doctors.

**Table 4:** Proportion of participants who responded to the various aspects of the role transition scale

Variables	Yes *n (%)*	No *n (%)*
Overall	51 (100)	0 (0)
BSN Roles		
Making Independent Rational Decisions/Autonomy	40 (78.4)	11 (21.6)
Independence from Doctors each time they make a patient decision	42 (82.4)	9 (17.6)
Working Collaboratively/Interdependence or Inter-disciplinary abilities	51 (100)	0 (0)
Individualized Care Planning	49 (96.1)	2 (3.9)
Holistic Care	48 (94.1)	3 (5.9)
Nature of work activities (high-level performance of skills)	51 (100)	0 (0)
Competence	45 (90.0)	5 (10.0)
Confidence (during care of patients)	50 (98.0)	1 (2)
Comfortable	49 (96.1)	2 (3.9)
Confident (in BSN roles)	49 (96.1)	2 (3.9)
Difference in Role Performance	50 (98.0)	1 (2)
Support		
Received Support	15 (29.4)	36 (70.6)
Understanding BSN Roles		
Other Staff on my unit Understood my BSN roles^a^	21 (41.2)	30 (58.8)
My Patients Understood my Roles as a BSN	22 (44)	28 (56.0)

*Note.* ^a^Eliminated while computing the reliability coefficient of the role transition score, due to internal inconsistence with other items in the scale

On the other hand, few participants (29.4%) reported having received support during the role transition process. Forty-one (41.2%) and forty-four (44%) percent thought that the other staff (their colleagues) and their patients did not understand their roles, respectively.

### Personal factors influencing role transition

Personal factors evaluated included sociodemographic characteristics, beliefs, and attitudes, experiences, motivators to pursuing the BSN, and knowledge and preparation. Pearson Product Moment Correlations indicated that moderate and positive correlations were obtained among the all-personal factors that statistically and significantly correlated with successful role transition (Table 5). In relation to the demographic characteristics of the participants, only one variable “performing any managerial role” weakly correlated with successful role transition (*r* = .297, *p* = .042). Among motivators to pursuing the BSN, job promotion (*r* = .331, *p* = .019) and career development (*r* = .371, *p* = .007) had moderate correlations; meanwhile, internal desire to self-development (*r* = .286, *p* = .042) had a weak correlation with successful role transition. Awareness of (*r* = .473, *p* = .000) and preparation (*r* = .346, *p* = .013) for the transition before one embarked on it had the strongest correlations with successful role transition.

**Table 5:** Bivariate Associations and Linear Regression Analysis of Personal factors that influence and predict Role Transition

Variables	*r*	A(*R*^*2*^)	*β*	*P*
Demographic Characteristics				
Managerial Role	.297^a^	.068	2.387	.042
Motivators				
Job Promotion.	.331^a^	.091	1.890	.019
Internal desire for Self-development	.286^a^	.063	4.833	.042
Career Development	.371^b^	.120	3.233	.007
Knowledge & Preparation				
Awareness of Role Transition.	.473^b^	.208	2.955	.000
Preparedness for Role Transition.	.346^a^	.102	1.990	.013
Cultural Beliefs and Attitudes				
Nurses/Doctors felt that I was doing doctors’ work	.172	.010	.838	.227
Other Nurses/Staff did not respect me as a BSN	.103	−.010	.472	.473
Experiences				
I have changed the way I practice nursing today as a BSN compared to when I was an RN	.336^a^	.095	1.396	.016
I was well prepared for my individualized care roles	.234	.035	1.971	.099
I did not feel pressure when I assumed more responsibilities, authority, and increased accountability	.357^a^	.109	1.384	.010
I was not stopped from carrying out certain tasks that belong to advanced roles of the BSN (RN) nurse	.374^b^	.122	1.487	.007
I did not feel guilt, emotional and or psychological dissatisfaction, and distress upon leaving my previous diploma RN roles	.349^a^	.104	2.079	.012
I felt that my new BSN roles did not distance me from patient-bedside care	.329^a^	.090	1.609	.018
I found it easier to delegate duties that I previously performed as an RN	.240	.058	.866	.093
My previous experience or knowledge on the complexity of the clinical setting made my transition easier	.308^a^	.077	1.170	.028
It was easier for me to let go of the way I practiced by RN roles	.394^b^	.138	1.961	.004
I felt like my BSN nurse roles were not confusing (ambiguous or vague)	.307^a^	.076	1.201	.028
As a BSN nurse, I felt I had achieved what took me back to school	.380^b^	.125	1.651	.008

*Note.* ^a^Correlation was significant at the 0.05 level (2 tailed) ^b^ Correlation was significant at the 0.01 level (2 tailed), **A (*R***^***2***^) adjusted R squared

It is also important to note that the positive experience of the participants had the largest number of variables that positively correlated with successful role transition. Those experiences with moderate correlations included finding it easier to let go of the way they previously practiced their RN roles (*r* = .394, *p* = .004), a feeling/perception that their BSN nurse roles were not confusing (*r* = .307, *p* = .028), and a feeling/perception that they had achieved what took them back to school (*r = .380, p = .008*). Additionally, a feeling/perception that their previous experience/knowledge on the complexity of the clinical setting had made their transition easier (*r* = .380, *p* = .008), a feeling/perception of no pressure when they assumed more responsibilities, authority, and increased accountability (*r* = .357, *p* = .010), and a feeling/perception that they were not stopped from carrying out certain tasks that belong to advanced roles of the BSN (RN) nurse (*r* = .374, *p* = .007) correlated moderately with successful role transition. The other experiences with moderate correlations with successful role transition included; a feeling/perception of no guilt, emotional and or psychological dissatisfaction, and distress upon leaving their previous RN roles (*r* = .349, *p* = .012); a feeling/perception of having changed the way they practiced nursing today as a BSN compared to when they were an RN (*r* = .336, *p* = .016), and a feeling/perception that their new BSN roles did not distance them from patient-bedside care (*r* = .329, *p* = .018). However, no perceived cultural beliefs that are ingrained in medical or nursing culture were found to correlate with successful role transition.

In bivariate linear regression, participants who performed managerial roles when compared with those who did not perform them, were 2.4 times (Table 5, under β) more likely to have a higher transition score and this variable explained about 6.8% of the variation in role transition (Table 5, **A**(***R***^***2***^)). Among motivators to pursuing the BSN, for every unit increase in the scores (range 1–3), on job promotion and career development, there was a predicted increase in role transition score by 1.890 and 3.233 times respectively, with career development having the largest explanatory power of 12% among motivators to pursuing the BSN. Participants who pursued the BSN because of an internal desire for self-development compared to others who upgraded to BSN on other reasons were 4.833 times more likely to have a higher transition score. For every unit increase in the scores, (range 1–4) of the participants being aware and prepared for role transition predicted 2.955 and 1.990 increase in role transition score and these variables had the highest explanatory power of 20.8 and 10.2%, respectively.

Under experiences, for every unit increase in the score (range 1–4), of the participant; finding it easier to let go of the way they practiced previously their RN roles, a feeling/perception that they had achieved what took them back to school, feeling/perception of no pressure when they assumed more responsibilities, authority, and increased accountability, a feeling/perception that they were not stopped from carrying out certain tasks that belong to advanced roles of the BSN (RN) nurse, and a feeling/perception of no guilt, emotional and or psychological dissatisfaction, and distress upon leaving their previous diploma RN roles, respectively, predicted a 1.961, 1.651, 1.384, 1.487, and 2.079, times increase in the role transition score. In addition, these variables under experiences had the highest explanatory power of 13.8, 12.5, 10.9, 12.2%, and 10.4% respectively. Variables with less than 10% explanatory power, under experiences included; a feeling/perception that their previous experience/knowledge on the complexity of the clinical setting made their transition easier (7.7%), a feeling/perception that their BSN nurse roles were not confusing, ambiguous or vague (7.6%), a feeling/perception of having changed the way they practiced nursing today as a BSN compared to when they were an RN (9.5%), and a feeling that their new BSN roles did not distance them from patient-bedside care (9%), predicted an increase in role transition score by 1.170, 1.201, 1.396, 1.609 times, respectively.

### Community factors that influence role transition

As a measure of community factors, this study investigated the forms and sources of support that RN to BSN nurses obtained or turned to during the difficult times of their transition. Bivariate analysis indicated that one variable (Doctors/physicians/consultants and other staff under the sources of support) significantly but weakly and negatively correlated with successful role transition (*r* = −.291, *p* = .038), Table 6). Other variables did not significantly correlate with successful role transition. In bivariate regression, doctors/physicians/consultants and other staff as a major source of support during their RN to BSN transition negatively influenced successful role transition by a factor of 1.395 and overall this variable explained a 6.6% variation in role transition.

**Table 6:** Bivariate associations and linear regression analysis of community factors that influence and predict role transition

Variables	*r*	A (*R*^*2*^)	*Β*	*P*
Forms of support obtained				
Information Support from Colleagues/Clinicians	.003	−.020	.018	.981
Mentorship from Nurses and Employer	−.084	−.013	−.013	.559
Financial Support from the Employer	.094	−.011	.541	.510
Sources of Support				
Employers/Nurse Managers	−.088	−.013	−.475	.539
Nursing Staff	−.172	.010	−.970	.229
Doctors/Physicians/Consultants and other Staff	−.291^a^	.066	−1.395	.038

*Note.* ^a^Correlation was significant at the 0.05 level (2 tailed), **A (*R***^***2***^) adjusted R squared

### Societal factors that influence and predict role transition

Barriers that may have impeded the transition to advanced roles were investigated as societal factors. In bivariate analysis, moderate, yet negative significant relationships were identified between successful role transition and barriers such as the BSN learning received during training was not applicable to their clinical setting (*r* = −.336, *p* = .016), and lack of support from colleagues (other BSNs & Doctors, (*r* = − 374, *p* = .007), Table 7). Unexpectedly, there was moderate, significant and positive relationship found between successful role transition and barriers such as BSN nurses being few to impact on the change of care (*r* = .343, *p* = .015), and lack of interest by other nursing cadres in learning what BSN nurses do (*r* = .337, *p* = .015). The majority of the other barriers did not significantly correlate with successful role transition.

**Table 7:** Bivariate Associations and Linear Regression Analysis of Societal Factors/Barriers that influence and predict Role Transition

Variables	*r*	A (*R*^*2*^)	*β*	*P*
Barriers				
BSN learning received during training not applicable to our Clinical Setting	−.336^a^	.095	−2.760	.016
Fewer BSN Nurses to Impact on the Change of Care	.343^a^	.099	1.622	.015
Lack of interest by other Nursing Cadres in Learning what BSN nurses do	.337^a^	.096	1.641	.015
Lack of Support from Colleagues (other BSN& Doctors)	−374^b^	.123	−1.799	.007
Reluctance to take up more Responsibilities	−.226	.032	−1.346	.110

*Note.* ^a^Correlation was significant at the 0.05 level (2 tailed) ^b^ Correlation was significant at the 0.01 level (2 tailed), **A (*R***^***2***^)^***:***^ adjusted R squared. Rationally, it was expected that these barriers would reduce successful role transition score

In bivariate linear regression, high rating (range 1–3) of participants that the BSN theory (what is taught in class) was not applicable to their clinical setting and lack of support from colleagues predicted a decrease in role transition score by 2.760 and 1.799 times and these had an explanatory power of 9.5 and 12.3%, respectively. On the other hand, the BSN nurses being few to impact on the change of care and lack of interest in learning what BSN nurses do by other nursing cadres, predicted an increase in role transition score by 1.622 and 1.641 times, with an explanatory power of 9.9 and 9.6%, respectively and these variables has no effect on successful role transition.

### Multivariate analysis of factors (under subscales) that predict role transition

This study compared the various subscales scores (motivators, knowledge and preparation, experiences, barriers, advanced role mastery, and support) with successful role transition. Pearson Product Moment Correlation revealed only three subscales (knowledge and preparation, experience, and advanced skills mastery) that significantly and positively correlated with successful role transition. Knowledge and preparation registered moderate correlations (*r* = .329, *p* = .020) while experience (*r* = .431, *p* = .002) and advanced skills mastery (*r* = .489, *p* = .000) had strong correlations.

Standard multiple regression methods were then applied to assess the influence of total scores of knowledge and preparation, experience, and advanced skills mastery (model with three variables) on successful role transition. The model accounted for 33% (*F* (3, 51*),*= 8.897, *p* = .000) of the variance in role transition and thus nearly 67% of the successful role transition is explained by factors not included in the model. In the final model, experience and advanced skills mastery were found to be statistically significant predictors of successful role transition.

Results suggest that higher scores on the role transitional experience subscale predicted a higher role transitional score by a factor of 0.151 (Table 8). Meanwhile, for every one-unit increase in the total scores of advanced skills; mastery, there is a predicted increase in role transitional score by a factor of 0.757 and this subscale made the strongest contribution in explaining the variation in successful role transition by 33.9% (see Table 8, under beta).

**Table 8:** Multiple linear regression analysis of factors (under subscales) that predict role transition

Variables					95.0% CI for *β*	
Sub-Scale	Β	S E	Beta	P	Lower	Upper
Knowledge & Preparation	.714	.504	.181	.163	−.301	1.728
Experience	.151	.054	.341	.007*	.042	.259
Advanced Skills Mastery	.757	293	.339	.013*	.166	1.348

*Note.* * Correlation was significant at the 0.05 level (2-tailed). Correlation was significant at the 0.01 level (2-tailed). Unstandardized coefficients (β) reported were significant at 0.05 or 0.01

## Discussion

Although previous studies on role transition among RN to BSN focused on evaluating this subject using the qualitative approach [6–10, 15, 16]. This study used the quantitative approach, guided by the transitional theory by Meleis et al. [16] to evaluate the factors that influence the transition to advanced roles. This study confirms some concepts of the theory in predicting successful role transition among RN to BSN, especially personal factors such as advanced skills mastery and experiences.

Broadly, this study found the majority of participants to have mastered the advanced skills of a BSN and had successfully transitioned into their BSN advanced roles. Mastery of the advanced skills of a BSN is a sign of a healthy transition as well as the ability of the BSNs to carry out their advanced roles [16]. Therefore, the study findings are much in line with other studies that found a positive association between the BSN prepared nurses and professional behaviors vital to patient care and patient safety [2, 3]. When we controlled for total scores on subscales of experience and knowledge and preparation, mastery of advanced skills significantly and positively influenced successful role transition. The finding also agrees with Nayda & Cheri [10], who stated that knowledge and skills expected of an RN, is a key factor in influencing a successful transition. However, these findings regarding role mastery and transition question the observations by health managers in Uganda, that some RN to BSN nurses find it difficult to change the way they practice nursing, once they returned to the workplace. Possible answers to this question could be first, the scope of practice and scheme of work for all nursing cadres in Uganda has not yet been implemented making judgment about the difference in practice very difficult. Second, even though the majority of the RN to BSN nurses thought that they were well prepared for the new RN to BSN roles, the Ugandan health care environment has not changed much since colonial days, to fit into their learning. Third, the failure to incorporate the RN to BSN into the government structure, pushing the new BSN to acquire employment outside the public sector, in order to be able to use their skills optimally and earn a better living, thus giving minimal attention to nursing care in their respective public hospitals, may also explain the health managers views. Relatedly, the low proportion of participants admitting to having critical thinking skills, leadership skills, and pro-activeness may provide some support for the health manager’s assertions, since they influence one’s performance. Aside from that, the ability of the majority of the RN to BSN nurses to make independent rational decisions as shown in this study is a major step in the right direction of improving the image of the nursing profession in Uganda and worldwide through the establishment of professional autonomy.

Results showed that BSN nurses who enrolled for the course with the desire to develop their career, or had an internal desire for self-development, or had a desire for job promotion were more likely to have a successful role transition and these findings were unique to this study. Despite the fact that no accessible study evaluated the influence of motivators to pursuing the BSN on successful role transition the above findings suggest that pull factors are key predictors of a successful role transition more than push factors.

Although this study did not find any medically ingrained cultural attitudes and beliefs that were associated with successful role transition, it did find that being aware and prepared for RN to BSN role transition before one embarked on it predicted an increase in the role transition score. However, in the final multivariate linear model, the total score for knowledge and preparation subscale did not significantly predict successful role transition, when controlled for experience and advanced skills mastery subscales/scores. Contrariwise, findings in previous studies showed that RN to BSN nurses’ who were unprepared for their new roles were more likely to have an unsuccessful role transition [7–9].

In this study, positive transitional experiences were found to influence successful role transition positively. This suggests that those participants who had positive experiences were more likely to have successfully transitioned. For instance, a feeling/perception that their previous experience or knowledge on the complexity of the clinical setting made their transition easier and that their new BSN roles did not distance them from patient-bedside care, were also found to positively influence successful role transition from RN to BSN. Nevertheless, previous authors [6, 7] found negative experiences were associated with unsuccessful role transition. Additionally, some previous authors although did not correlate role transitional experiences with successful role transition [6, 9, 10], they did find negative role transitional experiences among RN to BSN. One possible explanation for this discrepancy may be rooted in the qualitative research approaches used by previous researchers, whose data is non-generalizable. Nevertheless, some of these study findings relating to accountability and responsibility are consistent with previous literature for instance: Nayda & Cheri [10] reported that adjusting to the levels of accountability and responsibility was an influencing factor in successful role transition.

The study also discovered that participants who thought that the BSN learning received during training was not applicable to their clinical setting and thus posed as a barrier to successful role transition had a lower role transition score. This finding concurs with two previous authors [6, 10], who found out that a huge difference between the teaching institutions (ideal) and the working hospital environment (real), in terms of the recommended practices- prevented BSN nurses from carrying out some of their core duties such as holistic care (role dissonance).

Additionally, lack of support from colleagues as a barrier to role transition was found to predict a decrease in role transition score and consequently decrease one’s ability to change the way they practice nursing as a BSN. In fact, Nayda &Cheri [10] found out that the presence of a support system was a key factor that may influence successful transition after the BSN. Although a previous study [6], had indicated that clinical doctors were helpful in teaching new BSN skills when they observed that they were not confident and that this helped them to successfully role transition. To the contrary, this study found out that doctors/physicians/consultants and other staff as a major source of support during their RN to BSN transition negatively impacted on successful role transition. On a whole, this study suggests that personal factors may be influencing successful role transition as opposed to external factors (community and societal factors).

### Recommendations and implications of the study

#### Nurse managers/ employers/ professional regulatory body

Nurse managers and employers need to understand that there is a major difference in the role of the nurse when one attains a BSN, upon return to their workplace. BSN nurses can have a huge impact on patient outcomes if given an opportunity to practice at the level of their BSN training. The advanced skills they acquire in the BSN program and their ability to work as general care providers means that nurse managers have a variety of options to use the BSN nurses optimally at the workplace. For example, nurse managers can allocate BSNs in sensitive health units such as the intensive care units, in community centers, in management positions, in professional development of other nurses (such as in continuous medical/nursing education), and conducting quality assurance research geared toward improving the quality of care in hospitals and community centers among others. However, this requires that the employers/hospital managers provide enough and appropriate equipment and supplies/logistics, and health care environment to the nurses to be able to perform at a higher level. On the other hand, nurses should be able to return to their workplace, and practice all that they have learned: this requires that the hospitals have general and differentiated/specialized care units (such as intensive care units), that require special needs care, where the BSNs would implement their skills and thus not lose them.

In relation to findings that relate to motivators of role transition, it is incumbent upon employers, especially, in developing countries to recognize the nurses who have successfully transitioned in their roles, as a tool for encouraging professional development, motivation, and retention of RN to BSN nurses within the public service. However, recognition of BSNs may present with some costs and other issues like developing, and implementing a new scope of practice, a scheme of service, and filling up the workforce positions that may be created by the new scheme of service. Nevertheless, the implementation of the scheme of service could begin slowly and perhaps may be completed over years.

Since more than half of the sample thought that other staff (other health care professionals, other cadre nurses inclusive) did not understand their roles, it would be illogical/difficult for that other staff to support somebody to successfully role transition into their new roles which they do not understand. Therefore, nurse managers/employers and nurse educators have a role to sensitize and emphasize the roles of the BSN nurses right away during training, so that they can receive the necessary support from their colleagues and other health professionals.

#### Nurse educators

Nurse educators need to ensure that RN to BSN nurses are well trained and prepared for the role transition. During the preparations, nurse educators need to create awareness of the personal factors that RN to BSN nurses may encounter in their profession upon graduation and return to workplace. Nurse educators need also to regularly advocate and assess the learning environments, where their BSN students are sent for practicum to ensure they fit into their placement objectives.

In order to match theory to practice, nurse educators in liaison with the nursing professional regulatory body and employers should ensure that appraisal tools used during training, and upon return to workplace reflect the roles of a BSN, and are similar. Lastly, it is important for graduate nurses (other BSN nurses) who are already in practice to be empowered to supervise their colleagues as they begin their role transition.

#### Nurses, clinicians, and other staff

Nurses who have not yet transitioned and other professionals need to appreciate that RN to BSN nurses who return to the workplace are different in terms of their approach to health care provision. They also need support from nurse managers and other professionals to successfully, transition. This support may be in the form of information relating to the current clinical problems (in order to bridge the gap between theory and practice) and assisting them to master their advanced skills, for those who have not yet mastered them. Health professionals should also endeavor to create an enabling practice environment that cultivates positive experiences to the new RN to BSN, through changing some negative attitudes towards the image of the RN to BSN nurses and exercising retrain when commenting negatively about the weaknesses of new RN to BSN nurses. Instead, other health professionals should foster positive collegial and supportive relationships geared towards improving the image of the RN to BSN.

### Limitations of the study

First, this study had a small sample size, which limits the generalization of these study findings to the similar populations and this also limited this study analysis of the data (unable to meet 15 participants per variable for hierarchical multiple regression). Multiple linear regression would have helped to examine the relative influences of each of the different independent variables (in each subscale) on successful role transition and as well control for the confounding effects of each of those variables. Secondly, the data collected for this research was based on a convenience sample. Thus, proof of the participants meeting the inclusion criteria in some hospitals that did not have lists of RN to BSN nurses was very difficult. The findings of this study may also have been under or overstated, owing to the fact that participants self-reported the factors that influenced their own successful role transition. The lack of a scope of practice for BSN roles in Uganda may have also limited the participant’s understanding of the meaning of the research topic entitled- “transition from basic to advanced roles” and this may have affected how they responded to the research questions. Using literature on RN to BSN role transition and guided by the transitional theory, the researcher created this study tool and this tool may have not been very precise or clear to the study participants and may not have captured the essence of the difficulties in role transition. Above all, certain variables such as critical thinking were not measured using a standardized tool. Lastly, the tool failed to capture the role transition difficulties as perceived by the health managers, which might have enriched this study.

### Suggestions for further research

Even though the present study highlights some important factors that influence role transition, more research is needed using larger sample size and multiple methods (such as observations, focused groups). Most importantly, there is a need to compare and validate the study findings of the RN to BSN nurses with those of the other stakeholders such as health managers, employers, and other professionals among others. Another study may replicate the current study using a standardized tool to measure the study variables. There is also a need to find out the impact of BSN nurses on patient outcomes in a low resource setting like Uganda. A longitudinal study could also be undertaken focusing on successful role transition during the period when nurses’ start training in institutions up to when they return to the workplaces.

## Conclusion

On a whole, this study suggests that personal factors influence successful role transition more than external factors (community and societal factors). Key among the personal factors include advanced skills mastery and the role transitional experiences that predicted an increase in successful role transition. Therefore, this study calls for all nurse managers, educators, and clinicians among others, to focus on personal factors more than any other factors, so as to help RN to BSN nurses to role transition successfully at their respective workplaces.

## Additional files

Additional file 1: Questionnaire (PDF). This is the data collection tool that was used to collect survey data for this study. (PDF 174 kb)

Additional file 2: Data set (XLS). This is the data file where all the survey data collected was entered and used for analysis. (XLS 170 kb)

## Abbreviations

BSN: Bachelor of Science in Nursing
MOH: Ministry of Health-Uganda
MREC: Mulago National Referral Hospital Research Ethics Committee
RN: Registered Nursing
UNMC: Uganda nurse and midwives council
