# Supplementary material for: Factors that influence transition to advanced roles by RN to BSN nurses, in three selected hospitals of Central-Uganda
Source: BMC Nurs. 2019 May 6;18:18. doi: 10.1186/s12912-019-0345-z (PMC6501282; doi:10.1186/s12912-019-0345-z)
Supplement: Supplementary file 1 — Questionnaire (PDF). This is the data collection tool that was used to collect survey data for this study. (PDF 174 kb) [file 12912_2019_345_MOESM1_ESM.pdf]

Appendix A: Questionnaire

Questionnaire Number/code……/……/……………………………….
Hospital code……/………/…………………………………………….

N0.	Questions	Answers	Codes
**A. Socio demographic**			
A01	Gender.	1- Male<br>2- Female	□
A02	Age.	0-18-27 Years<br>1-28-37years<br>2-38-47 years<br>3-48-60years	
A03	Previous registration.	1-Nurse<br>2-Midwife<br>3. Registered both as Nurse and Midwife/double trained	
A04	Previous RN experience.	0-0-5years<br>1-6-10 years<br>2->10 years	
A05	Working experience after completing the BNS/Internship.	0- 6month- 2years<br>1-2.1-5years<br>2-5.1-10years	
A06	Previous unit of work before joining the BNS program.	1-Peadiatrics<br>2-Obstetrics<br>3-Medical<br>4-Surgery<br>5- Psychiatry<br>6-Any Other (Specify)…….	□
A07	Current unit of work.	1-Peadiatrics<br>2-Obstetrics<br>3-Medical<br>4-Surgery<br>5-Physchiatry<br>6-Any Other (Specify)…….	□
A08	Do you perform any managerial role?	1- Yes<br>2- NO	□

**B. Motivators to pursue BNS.**				
What motivated you to go for further nursing education (BNS)?<br>Of the following statements, please indicate how motivating they were in encouraging you to pursue a BNS ***(In this section, a list of statements are given upon which your opinion is needed on a rating scale of (1) Not Motivating (2) Somewhat Motivating, (3) Highly Motivating. You can Circle/Tick)***				
1. I wanted to obtain a better job with an improved pay /salary after acquiring the BNS.	**1**	**2**	**3**	
2. I wanted to secure my Job.	**1**	**2**	**3**	
3. I wanted to obtain a promotion.	**1**	**2**	**3**	
4. I felt competition from newer young graduates.	**1**	**2**	**3**	

5. Presence of support in terms of study scholarships.	**1**	**2**	**3**	
6. I was frustrated with the diploma RN roles (general nursing care roles).	**1**	**2**	**3**	
7. I felt my registered nurse roles were not respected by others.	**1**	**2**	**3**	
8. I wanted to work at a higher level and with increased responsibility, independence/autonomy, and accountability.	**1**	**2**	**3**	
9. I felt I needed to build my personal confidence in patient care	**1**	**2**	**3**	
10. I felt that at diploma (RN) level, there were limited opportunities for professional development.	**1**	**2**	**3**	
11. I felt there was a decline in employment opportunities for diploma RNs.	**1**	**2**	**3**	
12. I was confused with the scope of practice of diploma RN's	**1**	**2**	**3**	
13. I had an internal desire to develop myself/update my knowledge.	**1**	**2**	**3**	
14. There were opportunities to study, such as, distance learning.	**1**	**2**	**3**	
15. I wanted to develop my career.	**1**	**2**	**3**	
16. I wanted to experience/feel more satisfaction with my job.	**1**	**2**	**3**	
17. My employer wanted me to upgrade so that I may bring more skills to my work place.	**1**	**2**	**3**	
18. I saw the other BNSs succeed in their career, so I went back to school so that I may also succeed in my career.	**1**	**2**	**3**	
19. The hospital told us to go back and study because it was a requirement to work in my unit/hospital.	**1**	**2**	**3**	
20. Any other specify…………………………………………	**1**	**2**	**3**	
**C. Knowledge & Preparation for the BNS nurse Roles.**				
Before you came back to practice nursing as a BNS, did you know you had to change the way you practice nursing once you acquired a BNS and returned to this work place? *Mention your level of agreement with following statements about your knowledge and preparation for the BNS nurse roles.* ***(Circle /Tick 1=Strongly Disagree, 2=Disagree, 3=Agree, 4=Strongly Agree).***				
21. I was aware that I would change the way I practice nursing care after completing the BNS.*(changing from general nursing care to individualized care/care planning*).	**1**	**2**	**3**	**4**
Were you well prepared to change the way you would practice nursing once you returned to this work place?				
22. I was prepared to change the way I practice nursing care after completing the BNS.	**1**	**2**	**3**	**4**
**23.** If you Circled options ***3 or 4,*** for question 22, briefly explain how you prepared yourself for this change in practice. ………………………………………………………………………………………………… ………………………………………………………………………………………				
**D. Experience as a BNS nurse.**				
What were or what are your experiences of practicing nursing as a BNS, since you returned to this work place? *Below are some of the experiences a BNS nurse undergoes, mention your level of agreement with them, depending on how you experienced them* ***(Circle /Tick 1=Strongly Disagree, 2=Disagree, 3=Agree, 4=Strongly Agree)***				

24. I have the changed the way I practice nursing today as a BNS nurse compared to when I was an RN with diploma.	**1**	**2**	**3**	**4**
25. As a BNS nurse, It was not difficult-mentally or physically to Change the way I practice nursing *from bedside/general nursing care as an RN to individualized care/care plannin*g.	**1**	**2**	**3**	**4**
26. As a BNS nurse, I was well prepared for my individualized care/care planning roles.	**1**	**2**	**3**	**4**
27. As BNS nurse, I ***did not*** feel pressure when I assumed more responsibilities, authority, and increased accountability.	**1**	**2**	**3**	**4**
28. I felt higher satisfaction levels in my new BNS (RN) roles (individualized care/ care planning roles).	**1**	**2**	**3**	**4**
29. I ***was not*** stopped from carrying out certain tasks (such as comprehensive health assessment, care planning, holistic care giving among others) that belong to the individualized care/ care planning roles of the BNS (RN) nurse.	**1**	**2**	**3**	**4**
30. Higher patient load did not prevent me from practicing my BNS roles.	**1**	**2**	**3**	**4**
31. I ***did not*** feel guilt, emotional and or psychological dissatisfaction, and distress upon leaving my previous diploma RN roles.	**1**	**2**	**3**	**4**
32. I felt that my new BNS roles (of individualized patient care) ***did not*** distance me from patient-bedside care.	**1**	**2**	**3**	**4**
33. I found it ***easy to delegate duties*** that I previously performed as a Diploma RN.	**1**	**2**	**3**	**4**
34. My previous experience or knowledge on the complexity of the clinical setting ***made it easier*** for me to change the way I practice nursing as a BNS nurse.	**1**	**2**	**3**	**4**
35. Other Staff on my unit ***understood the roles*** that I had to play as a BNS nurse.	**1**	**2**	**3**	**4**
36. I felt ***My patients understood*** my roles as a BNS nurse	**1**	**2**	**3**	**4**
37. As a BNS nurse, the hospital environment and structures made it easier for me to change the way I practice/practiced nursing.	**1**	**2**	**3**	**4**
38. It was easier for me to let go of the way I practiced by RN roles (*previously before obtaining a BNS*).	**1**	**2**	**3**	**4**
39. As a BNS nurse, I wanted to remain on the same unit that I practiced on previously as an RN, before upgrading to a BNS.	**1**	**2**	**3**	**4**
40. I felt like my BNS nurse roles ***were not*** confusing (ambiguous or vague).	**1**	**2**	**3**	**4**
41. As a BNS nurse, I had ***no*** conflicts with other staff like doctors, clinical officer, and other nurses.	**1**	**2**	**3**	**4**
42. As a BNS nurse, I felt I had achieved what took me back to school..	**1**	**2**	**3**	**4**
43. Any other specify……………………………………………	**1**	**2**	**3**	**4**
44. There were some areas of my transition that were most difficult.	**1**	**2**	**3**	**4**

For question 44, If you circled option ***3 or 4***, briefly explain which areas of your transition that were most difficult upon resuming your work, after the BNS.<br>……<br>……<br>………………………………………………………………………………………..
**E. Barriers to Changing the way I practice Nursing as a BNS Nurse.**
**At this work place, what things may have stopped or discouraged you from practicing nursing as a BNS or what things encouraged you to practice nursing like a BNS nurse?**<br>*Of the following statements, please indicate the degree to which they may be affecting or affected the way you practiced/practice nursing as BNS nurse*. (***1= Little/No Influence, 2 = Moderate Degree of Influence, 3= High Degree of Influence)***

45. There is ***no scope of practice*** or a guide to show what a BNS nurse should do in care of patients *(how far a BNS nurse should go in terms of patient care*).	**1**	**2**	**3**	
46. I was ***not motivated*** to practice nursing as a BNS nurse because, when I returned after the BNS, the hospital did not promote me.	**1**	**2**	**3**	
47. What is taught in class at BNS ***is not applicable*** to our clinical setting and therefore it was difficult for me to implement it on ward once I returned to work.	**1**	**2**	**3**	
48. The ***environment at my work place*** did not allow me to change the way I practice nursing as BNS nurse.	**1**	**2**	**3**	
49. I ***failed*** to do care planning for individual patients, because we were few and the patient were many (high patient to nurse ratio).	**1**	**2**	**3**	
50. I ***do/did not do*** health assessment because doctors/clinical officers usually do it first &therefore there is no need for me to repeat it.	**1**	**2**	**3**	
51. ***I do not*** **document** patient care/care plan, because there are no papers for making nurses notes.	**1**	**2**	**3**	
52. ***I do not*** **document** patient care/care plan, because there no tool to document nursing care in the patient files.	**1**	**2**	**3**	
53. ***I do not do*** ***health assessment***, because there are no equipment such as B.P machines, thermometers, stethoscopes, etc, on ward for me to use.	**1**	**2**	**3**	
54. Nurses/doctors ***are not*** **interested** in the work of BNS nurses e.g the care plan, if you implement one, you implement it alone.	**1**	**2**	**3**	
55. We are **so few BNS nurses** to impact on the change of care on the ward.	**1**	**2**	**3**	
56. Nurses on the ward are ***not interested*** in learning what a BNS nurses does, because they are over burdened with patient work load and have no time to pay attention to it.	**1**	**2**	**3**	
57. There was ***lack of support*** from colleagues (other BNS& doctors) which could have helped me grasp my BNS nurse roles.	**1**	**2**	**3**	

58. There **was inadequate infrastructure** for learning or performing my new BNS roles (individualized) such as internet, books, private rooms or clinical space.	**1**	**2**	**3**	
59. There was **discrimination** against the BNS during promotions	**1**	**2**	**3**	
60. The other staff did not know or were confused about my roles.	**1**	**2**	**3**	
61. I had many conflicts with other staff (other nurse & doctors).	**1**	**2**	**3**	
62. I was reluctant to take up more responsibilities.	**1**	**2**	**3**	
63. There was a lack of refresher courses or **CME/CNE** in areas where I was **inadequately** trained.	**1**	**2**	**3**	
64. Nurses, physicians and doctors stopped me from performing (some of) my new BNS roles.	**1**	**2**	**3**	
65. Other nurses/staff did not respect me as a BNS	**1**	**2**	**3**	
66. Doctors seeing me perform a comprehensive health assessment, holistic care, and care planning among others threatened them.	**1**	**2**	**3**	
67. Nurses/doctors felt that I was doing doctors work.	**1**	**2**	**3**	
68. Any other barrier? Specify……………………………………	**1**	**2**	**3**	
**F. Support at My Work Place after Completing the BNS.**				
In this current work place, did you obtain any support from anybody that enabled you to change the way you practice nursing as a BNS?. *Of the following forms of support, please indicate how much you feel they were of much assistance to you in helping you change the way you practice nursing, as a BNS nurse.* ***(1= Little/No Assistance, 2 = Moderate Amount of Assistance, 3 =Much Assistance).***				
69. Information support from colleagues/Clinicians (staff taught me new skills).	**1**	**2**	**3**	
70. Mentorship from other nurses and employer.	**1**	**2**	**3**	
71. Financial Support from employer (through providing/lobbying for an increment in pay/promotion).	**1**	**2**	**3**	
Indicate how much you feel the following Categories of people were of much assistance to you in helping you change the way you practice nursing, as a BNS nurse. ***(1= Little/No Assistance, 2 = Moderate Amount of Assistance, 3 =Much Assistance)***				
72. Employers/Nurse managers.	**1**	**2**	**3**	
73. Nursing staff.	**1**	**2**	**3**	
74. Doctors/physicians/ consultants and other staff	**1**	**2**	**3**	
**G. Advanced Skills Mastery.**				
Have you mastered the following skills that are essential for you to practice nursing as a BNS? *On a scale of 1-4 rank your level of agreement with the following statements as a measure of mastery of the new skills required to practice your individualized care/care panning roles* ***(Tick/circle1=Strongly Disagree, 2=Disagree 3=Agree 4=Strongly Agree)***				
75. I pro-actively mobilize other nurse staff to solve emerging problems/issues related to patient care, within my unit.	**1**	**2**	**3**	**4**

76. I look out for or prevent patient's risks factors for illness or worsening of illnesses.	**1**	**2**	**3**	**4**
77. I communicate to patients better and confidently on any information related to their treatment.	**1**	**2**	**3**	**4**
78. I search out for nursing or medical interventions that have been proven to improve patient care outcomes and use it help improve care in my unit.	**1**	**2**	**3**	**4**
79. I weigh the various treatment options and nursing intervention before I administer them to the patient.	**1**	**2**	**3**	**4**
80. I am more knowledgeable in most patient illness now than when I was a diploma (RN).	**1**	**2**	**3**	**4**
**Role transition (Changing the way I Practice Nursing Today as a BNS).**				
How have you changed the way you practice nursing today as a BNS? On a scale of 1-4 rank your level of agreement with the following statements as one of the measure of the new ways of practicing your role **(*Tick/circle 1=Strongly Disagree, 2=Disagree, 3=Agree, 4=Strongly Agree)***				
81. Within ***<u>my scope of practice</u>*** as a BNS, I make some independent rational decisions related to nursing care for my patients without the doctor's permission or my superior's ***better than*** I did before as an RN.	**1**	**2**	**3**	**4**
82. I seek the doctor's permission each time I want to make a nursing decision for the patient's care.	**1**	**2**	**3**	**4**
83. I now work with other disciplines in care of patients, without fear, than before.	**1**	**2**	**3**	**4**
84. I make, monitor, and interpret patient care plans before I intervene**.**	**1**	**2**	**3**	**4**
85. I ensure my interventions are arrived at after a comprehensive assessment/I look at the big picture**.**	**1**	**2**	**3**	**4**
86. I now perform at a high level of skills function, more often, than a low level of skills when doing procedures, compared to when I was a diploma (RN**).**	**1**	**2**	**3**	**4**
87. I feel very competent while managing patients than before the BNS.	**1**	**2**	**3**	**4**
88. I confidently explain my assigned patient illnesses, and their progress, to doctors or my supervisors**.**	**1**	**2**	**3**	**4**
89. I feel more comfortable while managing patients than before the BNS**.**	**1**	**2**	**3**	**4**
90. While practicing my BNS roles, am very confident.	**1**	**2**	**3**	**4**
91. I believe the BNS program made a difference in the way I perform my roles today than when I was a Diploma (RN)	**1**	**2**	**3**	**4**

Give recommendations or any other comments about the way nurses change the way they practice nursing after the BNS (recommendations can be during ***training, internship, work place, to employers/government, nurse managers etc***)

…………………………………………………………………………………………………
…………………………………………………………………………………………………
…………………………………………………………………………………………………

……
……
……
……
……
……
…………………………

THANK YOU FOR YOUR COOPERATION & MAY GOD BLESS YOU.
